# Operation for the Removal of a Caseous Tumour of the Breast

**Published:** 1838-03-28

**Authors:** J. Randolph


*Operation for the removal of a Caseous Tumour of the Breast, by* J. Randolph, M. D.

*Wednesday,* 14*th March.*—Dr. Randolph prefaced the operation with some remarks on Cancer, for which we refer to No. 2, page 29. The patient was a black woman. He stated that the appearance of the tumour, together with the symptoms detailed by the patient, seemed to his colleagues and himself to resemble scirrhus. He proceeded to operate, present also Drs. Harris and Norris. A single incision was made with the bistoury, and the tumour dissected out with a scalpel. On examination, it proved to be a soft cheesy tumour, contained in a cyst.

*Wednesday,* 21*st.* The incision is nearly united, and the woman doing very well.

